# Microsporidia in Rodents—*Mus musculus*, *Rattus norvegicus,* and *Rattus rattus*—A Public Health Concern in the Canary Islands, Spain

**DOI:** 10.3390/ani15121695

**Published:** 2025-06-08

**Authors:** Sergio Llorens-Berzosa, Edgar Baz-González, Natalia Martin-Carrillo, Katherine García-Livia, Virginia Amaro-Ramos, Néstor Abreu-Acosta, Carmen del Aguila, Jordi Miquel, Román Pino-Vera, Estefanía Abreu-Yanes, Carlos Feliu, Fernando Izquierdo, Pilar Foronda

**Affiliations:** 1 Facultad de Farmacia, Universidad San Pablo-CEU, CEU Universities, Urbanización Montepríncipe, Boadilla del Monte, 28660 Madrid, Spain; ser.llorens.ce@ceindo.ceu.es (S.L.-B.); cagupue@ceu.es (C.d.A.); ferizqui@ceu.es (F.I.); 2 Departamento de Obstetricia y Ginecología, Pediatría, Medicina Preventiva y Salud Pública, Toxicología, Medicina Legal y Forense y Parasitología, Universidad de La Laguna (ULL), Avda. Astrofísico F. Sánchez, s/n, 38203 San Cristóbal de La Laguna, Spain; ebazgonz@ull.edu.es (E.B.-G.); nmartinc@ull.edu.es (N.M.-C.); kgarcial@ull.edu.es (K.G.-L.); alu0100773828@ull.edu.es (V.A.-R.); rpinover@ull.edu.es (R.P.-V.); 3 Instituto Universitario de Enfermedades Tropicales y Salud Pública de Canarias (IUETSPC), Universidad de La Laguna (ULL), Avda. Astrofísico F. Sánchez, s/n, 38203 San Cristóbal de La Laguna, Spain; nabreu@ull.edu.es; 4 Programa de Doctorado en Ciencias Médicas y Farmacéuticas, Desarrollo y Calidad de Vida, Universidad de La Laguna (ULL), Avda. Astrofísico F. Sánchez, s/n, 38203 San Cristóbal de La Laguna, Spain; 5 Nertalab S.L.U., 38001 Santa Cruz de Tenerife, Spain; esabya@gmail.com; 6 Departament de Biologia, Sanitat i Medi Ambient, Facultat de Farmàcia i Ciències de l’Alimentació, Universitat de Barcelona, Avda. Joan XXIII, s/n, 08028 Barcelona, Spain; jordimiquel@ub.edu (J.M.); cfeliu@ateneu.edu (C.F.); 7 Institut de Recerca de la Biodiversitat (IRBio), Universitat de Barcelona, Avda. Diagonal, 645, 08028 Barcelona, Spain

**Keywords:** Canary Islands, *Encephalitozoon* spp., *Enterocytozoon bieneusi*, microsporidia, rodents, zoonotic potential

## Abstract

Rodents are recognized as reservoirs of a wide range of pathogens, including microsporidia. The presence of microsporidia in the environment of mainland Spain and its islands has become increasingly known, as the number of studies has multiplied over time. The present study was conducted to determine the occurrence and diversity of microsporidia in three rodent species (*Mus musculus*, *Rattus norvegicus*, and *Rattus rattus*) in the Canary Islands, Spain. Ninety-three fecal samples were obtained from wild rodents on La Gomera and Gran Canaria Islands. Each sample was tested using Weber’s modified trichrome staining and immunofluorescence antibody tests (IFATs) against the *Encephalitozoon* genus and *Enterocytozoon bieneusi*. The microscopy-positive samples were subsequently analyzed using a nested polymerase chain reaction (PCR) followed by Sanger sequencing. The staining technique showed 38.7% (36/93) positivity, whereas the IFATs for *Encephalitozoon* spp. and *Ent. bieneusi* revealed 3.2% (3/93) and 6.5% (6/93) positivity, respectively. Finally, the nested PCR and nucleotide sequence analysis confirmed a 9.7% (9/93) occurrence of *Ent. bieneusi* and 17.2% occurrence (16/93) of different undetermined microsporidia species, whereas no *Encephalitozoon* spp. were detected. Seven different *Ent. bieneusi* genotypes were detected as follows: three known (AAE1, D, and SBM1) and four novel (GRE1, GRE2, LGE1, and LGE2), all of which belonged to Group 1. The results demonstrate, for the first time, that microsporidia are present in the rodent populations of the Canary Islands. Further studies are needed to determine the impact of the presence of microsporidia in rodents on the zoonotic transmission of these parasites.

## Simple Summary

Microsporidia are fungal parasites that infect invertebrate and vertebrate hosts, including humans. Mice and rats are known to be reservoirs of different pathogens, among them, microsporidia. These rodent species are distributed worldwide, including the Canary Islands (Spain), where there is no previous information on these pathogens in the rodent population. Therefore, the aim of this study was to evaluate the presence of microsporidia in the feces of wild rodents from two islands of the archipelago, La Gomera and Gran Canaria. This study was conducted using microscopic and molecular methods and confirmed the presence of microsporidia in mice and rats from the Canary Islands. Specifically, *Enterocytozoon bieneusi*, a species responsible for the majority of human microsporidiosis cases, with diarrhea being the most frequent symptom, was detected in rodents from both islands. These findings indicate, for the first time, that microsporidia are transmitted through rodent feces in the environment of the Canary Islands, which may have significant public health implications.

## 1. Introduction

Microsporidia are unicellular opportunistic parasites related to fungi [1]. Known as obligate intracellular parasites, these spore-forming eukaryotic organisms use a host’s cellular machinery to complete their life cycle. A wide range of invertebrate and vertebrate animals, including humans, can be infected by microsporidia through the ingestion of highly resistant spores [2,3]. Spore transmission occurs mainly via the fecal–oral pathway. The sources of infection are parasitized animals or humans, which act as agents of environmental contamination and the transmission of microsporidia spores [4,5] that can reach water [6] and even the food chain [7].

Currently, there are more than 220 genera and 1700 species of microsporidia [8], but only 17 species are known to be responsible for the diagnosis of microsporidiosis in humans [9], including *Enterocytozoon bieneusi* [10], *Encephalitozoon cuniculi*, *Encephalitozoon hellem*, and *Encephalitozoon intestinalis* [11,12], which are of major clinical interest. Although infection is possible in immunocompetent patients [13], microsporidia are frequently reported in immunosuppressed populations. The most common symptom of human microsporidiosis is chronic diarrhea, especially in *Ent. bieneusi* and *Enc. intestinalis* infections; however, microsporidia can also spread to the brain, lungs, liver, and other organs, leading to various pathologies [14]. Microsporidiosis is diagnosed using light microscopy (staining), electron microscopy, immunofluorescence, and molecular techniques [15]. Molecular typing tools based on a nucleotide sequence analysis of the internal transcribed spacer (ITS) region of the ribosomal RNA (rRNA) gene allow for identification at the genotype level, thus providing insight into the zoonotic potential. More than 500 genotypes of *Ent. bieneusi* are grouped into phylogenetic groups (Groups 1–15); the main groups with zoonotic or cross-species potential are Group 1 and Group 2, whereas the rest are considered host-adapted groups [16]. In the case of *Enc. cuniculi*, four genotypes with zoonotic potential are known (I, II, III, and IV), whereas *Enc. hellem* displays five different ITS genotypes (1A, 2A, 2B, 2C, and 2D), with genotype 1A being the most common reported in non-human mammal hosts [17].

Studies reporting the presence of microsporidia in immunocompetent wildlife have opened the door to their consideration as reservoirs for the transmission of zoonotic microsporidia [17,18,19]. In relation to this statement, rodents, such as rats and mice, are recognized as reservoirs of a large number of pathogens, including microsporidia [19,20,21,22].

In the Canary Islands, an archipelago of volcanic origin located approximately 100 km off the northwest coast of Africa, belonging to Spain, there are three alien murid species: the house mouse (*Mus musculus*), the brown rat (*Rattus norvegicus*), and the black rat (*Rattus rattus*). These species are distributed throughout the islands of the archipelago, both near human settlements and in rural areas [23,24]. Because of the lack of data on the presence of microsporidia in the rodents from this region and considering the previous reports of microsporidiosis in humans [13,25], this study aimed to investigate the occurrence and genetic diversity of microsporidia in wild rodents from the Canary Islands.

## 2. Materials and Methods

### 2.1. Ethical Agreement

The capture of rodents was authorized by the “Consejería de Transición Ecológica, Lucha contra el Cambio Climático y Planificación Territorial” (Gobierno de Canarias) (Expte. 2021/51732, 19-2022/1110092705). The capture of animals in protected areas (Parque Nacional de Garajonay) was approved by the “Cabildo Insular de La Gomera” (Expte. 4121/2022) and “Parque Nacional de Garajonay” (749.153, TELP/122.918). No protected or endangered species were included in this study.

### 2.2. Experimental Design

The following workflow was designed to study the presence of microsporidia. Briefly, several sampling campaigns were conducted on the islands of La Gomera and Gran Canaria, and fecal samples were collected from the intestines of trapped rodents. The presence of microsporidia was microscopically examined by modified trichrome staining and immunofluorescence assays. The samples with spore-compatible structures were selected for DNA isolation and nested polymerase chain reaction (PCR) performance. The PCR-positive samples were sequenced to determine the microsporidian species and/or genotypes.

### 2.3. Study Area, Samples Collection, and Preparation

**Figure 1:**
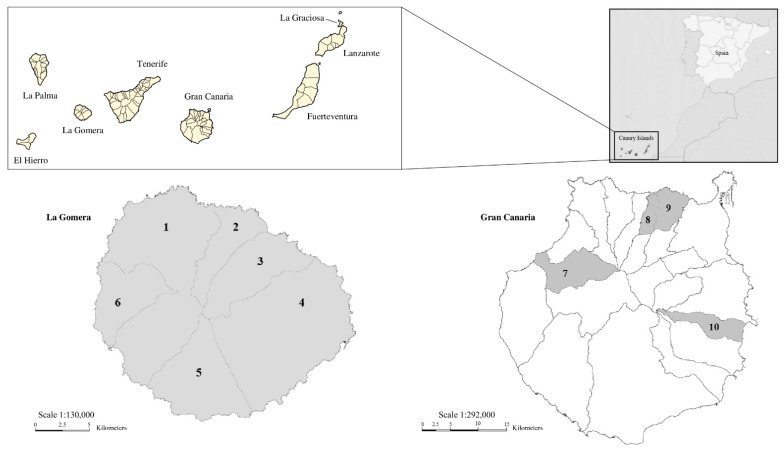
Map of the sampled areas on the islands of La Gomera and Gran Canaria (Canary Islands, Spain). The upper-right corner shows the Canary Islands, as they are related to Africa and mainland Spain. The upper-left panel shows the Canarian archipelago. The sampled municipalities of La Gomera Island are as follows: 1. Vallehermoso, 2. Agulo, 3. Hermigua, 4. San Sebastián de La Gomera, 5. Alajeró, and 6. Valle Gran Rey. Those of Gran Canaria Island are as follows: 7. Artenara, 8. Firgas, 9. Arucas, and 10. Ingenio. Adapted from Wikimedia Common (https://upload.wikimedia.org/wikipedia/commons/d/d1/Mapa_Canarias_municipios.svg, accessed on 10 March 2025; https://upload.wikimedia.org/wikipedia/commons/2/28/Islas_Canarias_%28real_location%29_in_Spain.svg, accessed on 10 March 2025), License CC BY-SA 3.0 (https://creativecommons.org/licenses/by-sa/3.0/, accessed on 26 May 2025), by which permission to copy, distributed, or adapt was established. Users: TUBS (https://commons.wikimedia.org/wiki/User:TUBS, accessed on 10 March 2025), Tintazul (https://commons.wikimedia.org/wiki/User:Tintazul, accessed on 10 March 2025). GRAFCAN and IDE Canarias (Source: Gobierno de Canarias (https://www3.gobiernodecanarias.org/medusa/mediateca/ecoescuela/?attachment_id=3322, accessed on 26 May 2025; https://www3.gobiernodecanarias.org/medusa/mediateca/ecoescuela/?attachment_id=3265, accessed on 26 May 2025), License CC BY-NC-ND 4.0 (https://creativecommons.org/licenses/by-nc-nd/4.0/, accessed on 26 May 2025), images modified with permission of the licensor.

From February 2022 to January 2023, rodent collection campaigns were conducted in six and four municipalities of La Gomera and Gran Canaria, respectively, following the study by Martin-Carrillo et al. [26] (Figure 1).

A total of 93 rodents were collected, 54 mice (*M. musculus*) and 39 rats (35 *R. rattus* and 4 *R. norvegicus*). In terms of location, 66 and 27 animals were trapped on the islands of La Gomera and Gran Canaria, respectively (Table 1). Finally, fecal samples from each animal were obtained from the intestine by dissection in the laboratory.

All the fecal samples were preserved in 1.5 mL tubes containing 2.5% (*w/v*) aqueous potassium dichromate solution (K_2_Cr_2_O_7_) (Merck, Darmstadt, Germany) and stored at 4 °C until processing.

### 2.4. Microsporidian Spores

*Encephalitozoon* spp. spores, used as the positive staining controls, were previously obtained from a culture with VeroE6 cells (ATCC CRL-1586): *Enc. intestinalis* (ATCC 50506), *Enc. cuniculi* (USP-A1) [27], and *Enc. hellem* (V-213) [28]. *Enterocytozoon bieneusi* spores were obtained from a fecal sample of a patient with HIV.

### 2.5. Microscopic Methods

#### 2.5.1. Light Microscopy: Modified Trichrome Stain

One thin smear was prepared from each sample and stained with Weber’s chromotrope stain (chromotrope 2R [Sigma-Aldrich, St. Louis, MO, USA], Fast Green [Sigma-Aldrich, St. Louis, MO, USA], and phosphotungstic acid [Sigma-Aldrich, St. Louis, MO, USA]) [29] and microscopically screened for microsporidia spores at 1000× magnification under a Leica DM750 microscope model ICC50 HD (Leica Microsystems, Heerbrugg, Switzerland). Each sample slide was examined for approximately 15 min. Samples with morphological and staining characteristics, such as ovoid or spherical and refractile structures, 0.8–4 µm in size, staining ranging from pink to reddish in color and, preferably, exhibiting a vacuole and a pinkish belt-like stripe indicating a polar tube, were considered eligible for molecular biology testing.

#### 2.5.2. Immunofluorescence Antibody Test

In parallel with the modified trichrome studies, immunofluorescence antibody test (IFAT) screening was conducted to detect the microsporidian spores of the genus *Encephalitozoon* and *Ent. bieneusi*.

A 1/4 dilution in Phosphate-Buffered Saline (PBS) 1× of a monoclonal antibody (mAb) of murine origin IgG2a isotype, designated 2C2, was used as the primary antibody because of its ability to detect a wall’s exospore and the developmental stages of *Encephalitozoon* species spores [30,31]. A polyclonal anti-mouse IgG antibody conjugated with Fluorescein-5-isothiocyanate (FITC) was used as the secondary antibody (Ref. F2012 Sigma-Aldrich) at 1/250 dilution in PBS with 0.5% Evans Blue (Merck). The samples were evaluated at 400×–1000× magnification under a microscope (model Eclipse Ni, Nikon, Tokyo, Japan). The UV light was filtered at a wavelength of 395–415 nm, and the observation light was 455 nm. Microsporidia positivity was determined by the presence of fluorescent spore-like structures 0.8–4 µm in size, and fluorescence was more intense in the wall area.

In a similar manner as described above, except for the use of a 1/50 dilution in PBS 1×, a species-specific mAb of murine origin IgG2a isotype, designated 6E52D9, was used as the primary antibody for the detection of *Ent. bieneusi* spores [32] because of its ability to bind to the spore wall of this species.

### 2.6. DNA Isolation

Following the modified trichrome staining and IFAT screening, DNA was extracted from 500 µL of each fecal sample with spore-compatible structures.

As a first step, the samples were washed twice with 500 µL sterile PBS 1× at room temperature and centrifuged at 13,500 rpm for 15 min to remove the potassium dichromate. A commercial FastDNA^®^ Spin Kit for Soil (MP Biomedicals, Solon, OH, USA) was used, according to the manufacturer’s instructions. The homogenizer FastPrep-24^TM^ 5G (MP Biomedicals, Solon, OH, USA) was used as a spore disruptor for 40 s at 6 m/s.

### 2.7. Nested PCR Amplification

Two nested PCR protocols were used for the molecular detection of microsporidia in the selected fecal samples. The first nested PCR was carried out using generic microsporidia primers (MSP1/MSP2A/MSP2B and MSP3/MSP4A/MSP4B) [33]. These primers target the partial sequence of the 16S rRNA gene, the complete ITS region, and the partial sequence of the 5.8S rRNA gene of a wide range of microsporidia species. Furthermore, an *Ent. bieneusi*-specific nested PCR was used (EBITS3/EBITS4 and EBITS1/EBITS2.4) [34], targeting the complete ITS region, and portions of the flanking 16S and 5.8S rRNA genes.

A total volume of 25 µL for each reaction was used in both nested PCR protocols, and each sample was tested using 1 µL of DNA template and 1 µL of dilution 1:1 in molecular biology water (VWR) for the first round of PCRs, whereas 1 µL of the primary PCR product was used in the second round. The nested PCRs were performed according to previously published protocols and conditions [35,36]. All the reactions were performed in an XP Cycler (Bioer Technology, Hangzhou, China), with negative and positive controls included in each round. The PCR products were examined by electrophoresis on 1.5% (*w/v*) agarose gels (Fisher Bioreagents, Madrid, Spain) stained with REALSAFE Nucleic Acid Staining Solution (20,000X, REAL, Durviz S.L., Valencia, Spain). The samples were considered positive if the nested PCR amplified a product within the expected size range for the generic microsporidia protocol (300–500-bp) and/or the specific protocol for *Ent. bieneusi* (390-bp) [34,37].

### 2.8. Sequencing and Phylogenetic Analysis

The nested PCR-positive products with the expected size were purified using ExoCleanUp FAST (VWR International, Haasrode, Belgium) and sequenced using the Sanger method at the University of La Laguna Sequencing Services (Servicios Generales de Apoyo a la Investigación, Universidad de La Laguna, Spain) or Macrogen Spain (Madrid, Spain). The resulting sequence chromatograms were inspected visually, compared using the Basic Local Alignment Search Tool (BLAST v2.16.0) in the GenBank database, and consecutively aligned using the ClustalW program included in the Molecular Evolutionary Genetic Analysis software (MEGA X v10.2.6, Hachioji, Japan).

The accuracy of the *Ent. bieneusi* nucleotide sequences was verified by sequencing two independent PCR products for each positive sample.

A phylogenetic tree was constructed using the Neighbor-Joining method, and the genetic distances were calculated using the Kimura 2-parameter model [38] with 1000 bootstrap replicates.

The nucleotide sequences generated in this study were deposited in GenBank under the accession numbers for *Ent. bieneusi* (PP873349–PP873357) and undetermined species (PP873358, PP883776–PP883779, and PV061650–PV061657).

### 2.9. Statistical Analysis

Clopper–Pearson 95% confidence intervals (95% CI) were calculated for each occurrence value. Fisher’s exact test was performed to evaluate differences in occurrence of microsporidia according to genus and host species, sex, and island of origin variables (IBM^®^ SPSS^®^ Statistics software V.29.0.1.0). *p*-value < 0.05 was considered statistically significant.

## 3. Results

### 3.1. Light Microscopy: Modified Trichrome Stain

**Figure 2:**
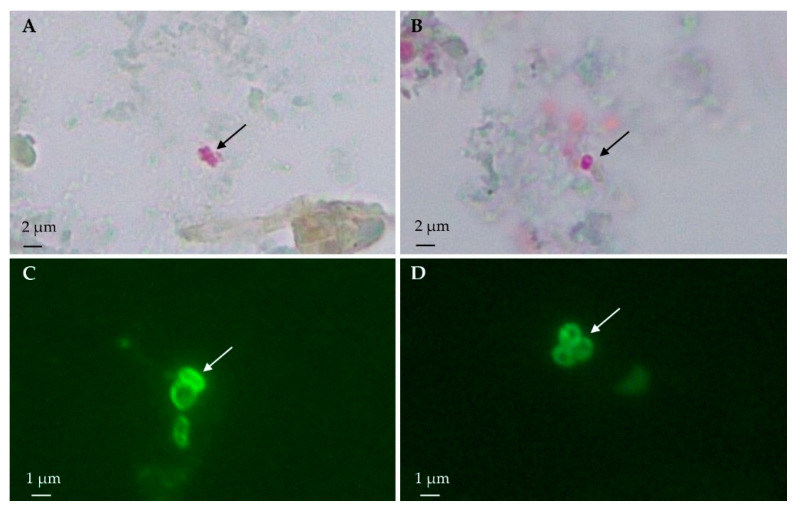
(**A**). Weber’s chromotrope stain of the intestinal contests of *Rattus norvegicus* showing three spore-compatible structures clustered together (**B**). *Mus musculus* showing a single spore-compatible structure (100×) (**C**). *Encephalitozoon* spp. spore-compatible structures were visualized by IFAT using mAb 2C2 (100×) in a sample from *Rattus rattus* (**D**). Cluster of spore-compatible structures with *Enterocytozoon bieneusi* visualized by IFAT using mAb 6E52D9 (100×) in a sample from *M. musculus*.

Spore-compatible structures were found in 38.7% (36/93) of the rodent samples stained with Weber’s chromotrope stain (Figure 2). The overall occurrence of microsporidia in *M. musculus* was 35.2% (19/54), whereas in the rats, it was 43.6% (17/39), specifically, 37.1% (13/35) in *R. rattus* and 100% (4/4) in *R. norvegicus* (Table 2).

Regarding the island of origin, the occurrence of microsporidia was 40.9% (27/66) on La Gomera and 33.3% (9/27) on Gran Canaria Island (Table 2). Spore-compatible structures were microscopically detected in eight of the ten municipalities sampled.

### 3.2. Immunofluorescence Antibody Test

The immunofluorescence antibody test analyses revealed spore-compatible structures with *Ent. bieneusi* in 6.5% (6/93) of the samples. Specifically, five samples from *M. musculus* (9.3%, 5/54) and one sample from *R. rattus* (2.9%, 1/35) were identified, whereas none were detected in any sample from *R. norvegicus* (0.0%, 0/4) (Table 2). In contrast, spore-compatible structures with the genus *Encephalitozoon* were found in three samples (3.2%, 3/93) (Figure 2). One sample was from *M. musculus* (1.9%, 1/54), two samples were from *R. rattus* (5.7%, 2/35), and none were from *R. norvegicus* (0.0%, 0/4). Only one sample from *M. musculus* showed structures compatible with both *Ent. bieneusi* and *Encephalitozoon* spp., suggesting a possible coinfection (Table 2).

### 3.3. Molecular Studies

After the microscopic analysis evaluation, of the 39 selected samples, 69.2% (27/39) yielded fragments of the expected sizes for a nested PCR for generic microsporidia, and 15.4% (6/39) yielded fragments of the expected size for *Ent. bieneusi*-specific nested PCR.

The sequence analysis revealed the presence of *Ent. bieneusi* in nine samples. Undetermined microsporidia species were identified in 16 samples based on a BLAST analysis because of the low identity observed (less than 95%) or because they were not long enough for a homology comparison with the reference sequences. *Encephalitozoon* spp. DNA was not detected using molecular techniques in any of the samples (Table 2).

### 3.4. Genotyping of Enterocytozoon bieneusi

A total of nine *Ent. bieneusi*-positive samples were detected; specifically, five were amplified with both nested PCR protocols, three samples were amplified only with the generic primers, and one sample was amplified only with the *Ent. bieneusi*-specific primers.

The ITS region analysis revealed the presence of three known genotypes—AAE1 (*n* = 1), D (*n* = 1), and SBM1 (*n* = 1)—and four novel genotypes—GRE1 (*n* = 1), GRE2 (*n* = 1), LGE1 (*n* = 3), and LGE2 (*n* = 1) (Table 3).

The ITS region of the novel genotypes GRE1 and GRE2 (242-bp in length) showed a deletion at position 53. Genotype GRE1 differed by two single nucleotide polymorphisms (SNPs) with respect to genotype AAE1 (accession nº: OQ646733) (G → T at position 144, T → C at position 209), and genotype GRE2 showed one SNP with respect to the latter genotype (C → A at position 110). In contrast, genotype LGE1 (243-bp) differed by one SNP from genotype HNR-VII (accession nº: MN931659) (G → A at position 10); genotype LGE2 (242-bp) presented with a deletion at position 53, and differed by one SNP from genotype AAE1 (accession nº: OQ646733) (T → C at position 151).

**Figure 3:**
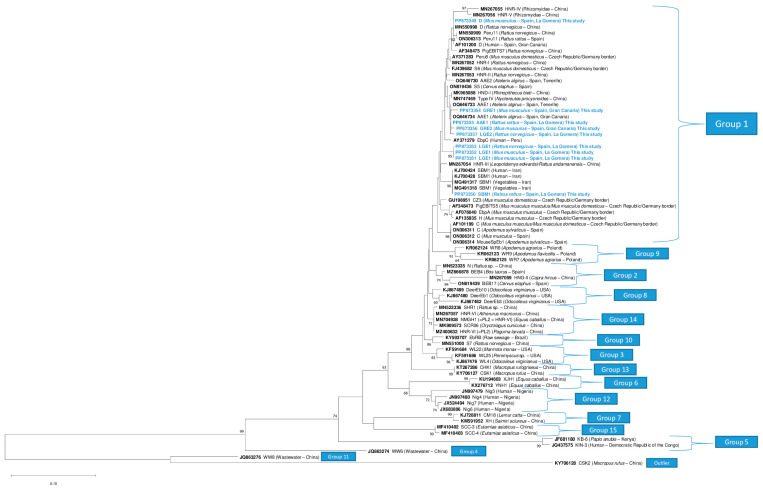
The phylogenetic relationships between the sequence of the internal transcribed spacer (ITS) region of the ribosomal RNA gene of the *Enterocytozoon bieneusi* genotypes obtained in this study and the sequences of known genotypes deposited in GenBank. The Neighbor-Joining method was used based on the genetic distance calculated using the Kimura 2-parameter model. Reference sequences from each phylogenetic group of *Ent. bieneusi* genotypes (Groups 1–15) were used. The accession numbers are shown in bold, and information regarding the host species and origin is shown in parentheses. There were a total of 240 positions in the final dataset.

The phylogenetic analyses showed that novel genotypes GRE1, GRE2, and LGE2, identified in rodents, were clustered together with genotype AAE1, identified in the North African hedgehog (*Atelerix algirus*) in the Canary Islands, whereas the isolates identified as the novel genotype LGE1 were clustered in an independent clade. All belonged to phylogenetic Group 1. The known genotypes (AAE1, D, and SBM1) were grouped with their respective reference sequences (Figure 3).

### 3.5. Geographical Distribution

*Enterocytozoon bieneusi* was found in both islands, La Gomera and Gran Canaria. On La Gomera Island, the known genotypes AAE1 and SBM1 were found in *R. rattus* (*n* = 1 each) and genotype D in *M. musculus* (*n* = 1). On this island, the novel genotype LGE1, the most frequently detected genotype, was found in *R. norvegicus* (*n* = 1) and *M. musculus* (*n* = 2), whereas the novel genotype LGE2 was only found in *R. norvegicus* (*n* = 1) (Table 3). In contrast, the novel genotypes GRE1 and GRE2 were detected on Gran Canaria Island, both in *M. musculus* (*n* = 1 each) (Table 3).

*Enterocytozoon bieneusi* showed a wide distribution on La Gomera Island, covering a total of three out of six municipalities on the island, namely Hermigua, San Sebastián de La Gomera, and Vallehermoso (Table 4). On the contrary, this species was detected only in one municipality of Gran Canaria. Of the seven *Ent. bieneusi* genotypes identified in this study, the most frequently detected was LGE1 (33.3%, 3/9). This novel genotype was exclusively found in the municipality of Hermigua (La Gomera). On the other hand, the occurrence of the other six genotypes found was 11.1% (1/9) each. The novel genotype LGE2 and genotype SBM1 were also found in the municipality of Hermigua. Genotypes D and AAE1 were detected in Vallehermoso and San Sebastián de La Gomera, respectively. With respect to the distribution of the genotypes on Gran Canaria, the novel genotypes GRE1 and GRE2 were found only in the municipality of Ingenio (Table 4).

Finally, other microsporidian sequences belonging to different undetermined species were detected in Agulo (*n* = 2), Alajeró (*n* = 1), Hermigua (*n* = 7), and Vallehermoso (*n* = 3) on La Gomera, and Ingenio (*n* = 3) on Gran Canaria Island (Table 4).

### 3.6. Statistical Results

No significant differences were found between the occurrence of *Ent. bieneusi* and any of the variables studied using Fisher’s exact test. *Rattus norvegicus* showed significant differences in the overall occurrence of microsporidia (*p* < 0.05), which was determined by observing a higher occurrence value with respect to the other rodent species analyzed (Supplementary Material: Table S1).

## 4. Discussion

This study presents the first detection and genotyping of microsporidia in rodents from the Canary Islands (Spain), and suggests that these animals may serve as reservoirs for microsporidia in the environment. The moderate occurrence and widespread distribution of the *Ent. bieneusi* genotypes with zoonotic potential raise potential public health concerns for the Canarian archipelago.

The intestinal contents of the rodents from La Gomera and Gran Canaria Islands were examined using Weber’s modified trichrome staining and IFATs for screening, with molecular methods as a confirmatory approach. Staining techniques, such as Weber’s modified trichrome, have proven very useful in detecting microsporidian spores and have been widely used for decades in parasitology laboratories [29]. Nevertheless, the small size of microsporidia may result in their misidentification with other microorganisms, such as small yeasts, bacteria, or fecal debris [39,40], leading to their potential overestimation. Conversely, samples with a low spore load may result in an underestimation of the presence of microsporidia [41]. Therefore, it is advisable to complement the classic staining methods with other detection techniques. Accordingly, IFATs and molecular techniques have been shown to be convenient and robust approaches for the detection and identification of microsporidian spores of both human and animal origin [30,31,35].

The presence of microsporidia spores detected in this study by classical staining (38.7%, 36/93) differed slightly from the overall occurrence obtained according to the two nested PCR protocols (29.0%, 27/93). This kind of discrepancy has been reported in other studies and could be the result of the spontaneous extrusion of spores [42], microscopic misidentifications [39], potential PCR inhibitors coeluted with the DNA from fecal samples [43], or the presence of microsporidia species that were not amplified with the primers used in this study [35].

In contrast, the overall occurrence of a positive IFAT (8.6%, 8/93) was the lowest among the three detection methods used. This low occurrence may be attributed to the high specificity of the mAbs used. As opposed to the modified trichrome method, which is able to stain spores of different species by staining the chitin wall that covers them, the antibodies used in this study are tools developed for the specific detection of spores of the genus *Encephalitozoon* [30,31] or the species *Ent. bieneusi* [32]. Another factor that could have influenced our results is the possible degradation of or even some modification in the structural conformation of the antigens present in the spore wall, which could have affected the specificity of antigen–antibody binding and, thus, the sensitivity of the technique due to the lag between sample collection and analysis.

Eight samples (8.6%, 8/93) were initially identified by IFATs as potentially containing spores of *Ent. bieneusi* (6.5%, 6/93) or species of the genus *Encephalitozoon* (3.2%, 3/93). Molecular analyses confirmed the presence of microsporidia in seven of the eight samples. Nested PCRs and sequencing confirmed the presence of *Ent. bieneusi* (*n* = 3) and undetermined microsporidia species (*n* = 1) in the IFAT-positive samples using an mAb specific for *Ent. bieneusi*, whereas two samples could not be molecularly confirmed. Regarding the samples identified as positive using the mAb against the *Encephalitozoon* genus, only undetermined microsporidia species were detected (*n* = 3). Considering these results, the IFAT analyses showed a good correlation between the immunofluorescence and the PCR-based assays, which suggests the possibility of cross-reactions with undetermined species of microsporidia, as has been seen for other monoclonal antibodies reacting with insect microsporidia [44].

Rodents have been extensively studied as reservoirs of microsporidia in various regions of the world, including the American continent [45], Asia [46], and Europe [47]. In Europe, the occurrence of *Ent. bieneusi* in wild rodent feces has ranged from 1.07% (3/280) in Slovakia [48] to 51.3% (98/191) in Poland [47]. In mainland Spain, the wild rodent population has shown an overall occurrence of 2.6% (12/490) for *Ent. bieneusi* [49], lower than the 9.7% (9/93) observed in this study. Vioque et al. [49] analyzed pooled samples of several species of micromammals belonging to the families Muridae, Cricetidae, Soricidae, and Sciuridae, but only three species of murids were positive for *Ent. bieneusi*: *Apodemus sylvaticus* (25%, 8/32), *M. musculus* (25%, 1/4), and *R. rattus* (14.3%, 3/21). More recently, *Ent. bieneusi* (*n* = 2) and *Enc. hellem* (*n* = 1) have been found sporadically in urban rats in the city of Valencia [50], demonstrating the circulation of human-pathogenic microsporidia in the rodent populations of Spain. On the other hand, no positive cases of *Ent. bieneusi* (0.0%, 0/64) were detected in free-living sympatric rats (*Rattus* spp.) captured in a zoo conservation center in Córdoba, in southern Spain [51]. In addition to rodents, *Ent. bieneusi* has been detected in wildlife in Spain, specifically in the European rabbit (*Oryctolagus cuniculus*) (0.8 [3/384]–14.3% [1/7]), red fox (*Vulpes vulpes*) (9.2 [8/87]–14.3% [1/7]), beech marten (*Martes foina*) (11.1%, 1/9), European badger (*Meles meles*) (23.2%, 16/69), boar (*Sus scrofa*) (0.8 [3/359]–2.1% [3/142]), red deer (*Cervus elaphus*) (1.5%, 5/329), Iberian lynx (*Lynx pardinus*) (3.1%, 7/224), North African hedgehog (*A. algirus*) (47.2%, 17/36), and Iberian wolf (*Canis lupus*) (1.3%, 3/225) [5,37,52,53,54,55,56,57]. *Encephalitozoon* spp. have only been identified in sporadic cases in the European rabbit, the Iberian hare (*Lepus granatensis*), and the Iberian wolf [35,56,58,59].

Our results are in accordance with those of previous studies, such as the meta-analysis by Taghipour et al. [60], which reported a microsporidia global occurrence of 14.2% in rodents, with *Ent. bieneusi* being the microsporidia most frequently detected by PCR (13.6%). The sequence analysis of the ITS region revealed the presence of seven *Ent. bieneusi* genotypes in this study, showing relatively high genetic diversity, in agreement with previous studies based on wild rodents [61]. Five genotypes were detected on La Gomera Island (AAE1, D, LGE1, LGE2, and SBM1). Genotype AAE1 was first identified in the North African hedgehog (*A. algirus*) in Tenerife and Gran Canaria [37]. Therefore, its presence in *R. rattus* on La Gomera indicates the wide distribution and potential cross-species transmission of this genotype in the archipelago. Genotype D has been reported as the most frequent genotype in rodent populations [60] and one of the most common worldwide [62]. This genotype has been previously reported in rabbits, red foxes [5], cats (*Felis catus*) [63], and Iberian lynxes [55] in mainland Spain, but this study constitutes the first time it has been found in rodents in this country to date [49]. Interestingly, cases of human microsporidiosis caused by this genotype have been identified in transplant recipients from Gran Canaria Island [25]; however, in this study, it was only found in *M. musculus* on La Gomera and there were no positive cases for this genotype on Gran Canaria, possibly because of the limited sample size, suggesting potential zoonotic transmission.

The genotype detected in *R. rattus* on La Gomera (accession nº: PP873351) showed a 100% identity with respect to the sequences previously identified in patients with cancer (accession nº: KJ700424) and HIV (accession nº: KJ700428 and KJ700432) in Iran, and was misidentified as genotype EbpC (cited as genotype E) [64]. This genotype was recently found in feral cats on the Canary Islands and renamed as genotype SBM1 (Baz-González et al.—unpublished). In the phylogenetic tree (Figure 3), these sequences are clearly different from the proper genotype EbpC (also named as “Peru4”, “WL13”, or “E” in other studies) (accession nº: AY371279, AY237221 and AF076042) [65]. Regarding the epidemiology of genotype SBM1, it has only been detected in human patients, wastewater, and vegetable samples in Iran [64,66], and more recently in feral cats on the Canary Islands (Baz-González et al.—unpublished), suggesting a rodent origin for this genotype.

The novel genotype LGE1 was detected in *M. musculus* and *R. norvegicus*, suggesting a cross-species transmission event between the mouse and rat populations on the island. Only one case of genotype LGE2 was reported, namely in *R. norvegicus*, all of them in the Hermigua municipality, suggesting the presence of different genotypes within the rodent population in this municipality. Two novel genotypes were detected on Gran Canaria Island, and GRE1 and GRE2 were detected in *M. musculus*. The previous genotypes detected in rodents in Spain are genotype C, Peru11, and MouseSpEb1, which have never been reported before in the archipelago [49].

The positive sample for genotype GRE1, and two of the three positive samples for genotype LGE1, all of which corresponded to *M. musculus*, showed *Ent. bieneusi* spore-compatible structures under the IFAT analyses, suggesting active spore shedding. Therefore, this host species is the most frequent carrier of zoonotic *Ent. bieneusi* genotypes on the Canary Islands and may contribute to environmental pollution by spore shedding in feces.

Regarding the genus *Encephalitozoon*, previous studies have demonstrated the presence of *Enc. cuniculi* (genotype I) in wild mammals on the Canary Islands, including the European rabbit [35] and the North African hedgehog [37] on the island of Tenerife, demonstrating its presence in the archipelago. Nevertheless, the presence of species belonging to the genus *Encephalitozoon* could not be confirmed using the molecular techniques in this study. The discrepancy between the different diagnostic methods used could be due to the possibility of cross-reactivity with other microsporidia species, as mentioned above. Microsporidia spores closely related to *Vairimorpha lymantriae*, *Nosema ceranae*, and *Sporanauta perivermis* by nucleotide sequence analyses were identified in the IFAT-positive samples using mAb against the *Encephalitozoon* genus (Supplementary Material: Table S2). Considering that these species, together with *Encephalitozoon* spp., form the recently described phylogenetic clade Nosematida [67], the possibility of cross-reactivity with undetermined microsporidia species closely related to the genus *Encephalitozoon* is supported. It is also possible that autofluorescence, as well as the presence of other microsporidia spores with epitopes that could be recognized by the antibodies used in this study, might have contributed to the observed discrepancies [44]. Although cases of *Enc. cuniculi*, *Enc. intestinalis*, and *Enc. hellem* have been described in *M. musculus* and *Rattus* spp. in the literature [48,50,68], the absence of these species is consistent with other studies carried out on fauna from mainland Spain, namely the Iberian lynx [55].

Finally, other microsporidia species were molecularly confirmed in 17.2% (16/93) of the animal samples, which is in agreement with previous reports on its presence in rabbits (10.0%, 5/50) and hedgehogs (9.1%, 3/33) on Tenerife Island [35,37]. Undetermined species were detected in 11.1% (6/54) of the studied mice and 25.6% (10/39) of the rats. The occurrence of samples showing undetermined microsporidia species was similar on both islands: 11.1% (3/27) on Gran Canaria and 19.7% (13/66) on La Gomera. Only four nucleotide sequences were sufficiently long for homology comparisons and had more than 90% of coverage. Isolate 502P (accession nº: PP873358) obtained from *R. norvegicus* showed a relatively high identity with the microsporidia species infecting invertebrates: *Anncaliia meligethi* (accession nº: AY894423) isolated from the pollen beetle (*Meligethes aeneus*) and *Anncaliia azovica* (accession nº: KY288065) isolated from *Niphargogammarus intermedius* [69,70]. Isolate RGB9 (accession nº: PV061652) obtained from *M. musculus* showed more than a 90% identity with *Tubulinosema pampeana* (accession nº: KM883008) isolated from the South American bumble bee (*Bombus atratus*) [71]. Finally, isolate 309d (accession nº: PV061650) obtained from *M. musculus* was identified as closely related to *Vairimorpha necatrix* (accession nº: JX213791) isolated from the moth *Lacanobia oleracea*, suggesting that microsporidia infecting invertebrate hosts passes through the gastrointestinal tract [35]. Undetermined species have been detected in other studies conducted in mainland Spain, with reports of their presence in animals and different water sources, suggesting their wide presence in the environment and their possibly belonging to species that infect insects or other hosts with limited or no zoonotic/cross-species transmission risk [5,72].

The specific habitat conditions, social behavior, and feeding habits of animals may also play a key role in facilitating the transmission of parasites between hosts and in maintaining the presence of microsporidia in an environment [21,73]. Studying the possible presence of microsporidia in free-living animals is crucial for public health. Over time, different studies have shown that certain species or genotypes of microsporidia that were previously thought to be specific to particular animal hosts have subsequently been found to infect humans, raising concerns about animals’ status as reservoirs for this parasite and the zoonotic nature of the microsporidia [16,18,74,75,76].

The present study has some limitations. Light microscopy and immunofluorescence techniques confirmed the role of rodents as shedders of microsporidia spores. However, by examining only the microscopy-positive samples by nested PCRs, the occurrence of microsporidia may have been underestimated, as the samples that may have been microscopy-negative but PCR-positive were not detected [77]. Microsporidia spores are eliminated by various biological fluids of a host, not only the feces, so it is advisable to examine other types of tissue from different organs (mainly the spleen, kidneys, and brain) [47,78]. The selected primers were useful for genotyping human-pathogenic microsporidia, but for undetermined microsporidia species, primers targeting conserved regions and amplifying larger fragments should be used to elucidate a possible origin and perform more complete phylogenetic analyses, especially of the partial/complete large-subunit region and small-subunit ribosomal RNA gene [67].

## 5. Conclusions

A significant overall rate of the presence of microsporidia in the rodent population of the Canary Islands is reported. This survey shows that different species of microsporidia species circulate widely among the wild rodents of the Canarian archipelago. The detection of *Ent. bieneusi* suggests a potential zoonotic risk from *M. musculus*, *R. rattus*, and *R. norvegicus*. The phylogenetic analyses suggest a zoonotic or cross-species transmission potential for the genotypes identified in this study. The relatively high occurrence of microsporidia detected in these free-ranging animals and the large number of rodents living in this region, in addition to the detection of active spore shedding, indicate that they may be a significant source of environmental microsporidian contamination posing a public health risk. Expanding our knowledge about the presence of microsporidia in globally distributed animals, such as rodents, may improve our understanding of their distribution in the archipelago. A larger study covering more geographical regions of the Canary Islands would be advisable to expand our knowledge about the role of rodents as a reservoir for zoonotic microsporidia.

## Figures and Tables

**Table 1:** Origin of the rodent host species analyzed in this study.

Host Species	Island	Sample Size (*n*)
*Mus musculus*	La Gomera	33
	Gran Canaria	21
	Subtotal	54
*Rattus rattus*	La Gomera	29
	Gran Canaria	6
	Subtotal	35
*Rattus norvegicus*	La Gomera	4
	Subtotal	4
	Total	93

**Table 2:** Results of microsporidia analysis in wild rodent fecal samples from the Canary Islands using modified trichrome staining, immunofluorescence antibody tests, and nested PCR.

Host Species	Modified Trichrome Stain (%) (+/*n*) [95% CI]	IFAT *Enceph.* (%) (+/*n*) [95% CI]	IFAT *Ent. bieneusi* (%) (+/*n*) [95% CI]	Generic Nested PCR (%) (+/*n*) [95% CI]	*Ent. bieneusi* Nested PCR (%) (+/*n*) [95% CI]	Microsporidia Species (*n*)	Occurrence (%) (+/*n*) [95% CI]
*Mus* *musculus*	35.2% (19/54) [22.7–49.4]	1.9% (1/54) [0.05–9.9]	9.3% (5/54) [3.1–20.3]	24.1% (13/54) [13.5–37.6]	5.6% (3/54) [1.2–15.4]	*Ent. bieneusi* (5) Undetermined (6)	9.3% (5/54) [3.1–20.3] 11.1% (6/54) [4.2–22.6]
*Rattus* *rattus*	37.1% (13/35) [21.5–55.1]	5.7% (2/35) [0.70–19.2]	2.9% (1/35) [0.07–14.9]	28.6% (10/35) [14.6–46.3]	2.9% (1/35) [0.07–14.9]	*Ent. bieneusi* (2) Undetermined (8)	5.7% (2/35) [0.70–19.2] 22.9% (8/35) [10.4–40.1]
*Rattus* *norvegicus*	100% (4/4) [39.8–100]	0.0% (0/4) [0.0–60.2]	0.0% (0/4) [0.0–60.2]	100% (4/4) [39.8–100]	50.0% (2/4) [6.8–93.2]	*Ent. bieneusi* (2) Undetermined (2)	50.0% (2/4) [6.8–93.2] 50.0% (2/4) [6.8–93.2]
Total	38.7% (36/93) [28.8–49.4]	3.2% (3/93) [0.67–9.1]	6.5% (6/93) [2.4–13.5]	29.0% (27/93) [20.1–39.4]	6.5% (6/93) [2.4–13.5]	*Ent. bieneusi* (9) Undetermined (16)	9.7% (9/93) [4.5–17.6] 17.2% (16/93) [10.2–26.4]

**Table 3:** Diversity and frequency of *Enterocytozoon bieneusi* genotypes identified in wild rodents from the Canary Islands.

Host Species	Genotype	Frequency (%) (+/*n*)	GenBank Reference Sequence (Accession nº)	Sequence Obtained in This Study (Accession nº)
	GRE1	20% (1/5)	-	PP873354
*Mus musculus*	GRE2	20% (1/5)	-	PP873356
	LGE1	40% (2/5)	-	PP873351 and PP873352
	D	20% (1/5)	MN747468	PP873349
*Rattus rattus*	AAE1	50% (1/2)	OQ646734	PP873355
	SBM1	50% (1/2)	MG491317	PP873351
*Rattus norvegicus*	LGE1 LGE2	50% (1/2)	-	PP873353
		50% (1/2)	-	PP873357

**Table 4:** Geographical distribution of microsporidia species and *Enterocytozoon bieneusi* genotypes identified in wild rodents from La Gomera and Gran Canaria, Canary Islands, Spain.

Island	Host Species	Municipality	Sample Size (*n*)	Modified Trichrome Stain (+)	IFAT *Enceph.* (+)	IFAT *Ent. bieneusi* (+)	Generic Nested PCR (+)	*Ent. bieneusi* Nested PCR (+)	*Ent. bieneusi* Genotype(s) (*n*)	Undetermined Microsporidia Species (*n*)
La Gomera	*Mus musculus*	Agulo	5	2	0	0	2	0	-	2
		Alajeró	3	1	0	0	1	0	-	1
		Hermigua ^1^	14	7	1 ^2^	4 ^2^	6	2	LGE1 (2)	2
		San Sebastián de La Gomera	6	1	0	0	0	0	-	-
		Vallehermoso	5	2	0	0	1	1	D (1)	1
	*Rattus rattus*	Agulo	4	1	0	0	0	0	-	-
		Alajeró	2	0	0	0	0	0	-	-
		Hermigua	12	5	1	1	5	1	SBM1 (1)	4
		San Sebastián de La Gomera	4	2	0	0	1	0	AAE1 (1)	-
		Vallehermoso	3	1	0	0	1	0	-	1
		Valle Gran Rey	4	1	0	0	0	0	-	-
	*Rattus norvegicus*	Hermigua	3	3	0	0	3	2	LGE1 (1) LGE2 (1)	1
		Vallehermoso	1	1	0	0	1	0	-	1
Gran Canaria	*Mus musculus*	Artenara	2	2	0	0	0	0	-	-
		Arucas	1	0	0	0	0	0	-	-
		Firgas	1	0	0	0	0	0	-	-
		Ingenio ^1^	17	4	0	1	3	0	GRE1 (1) GRE2 (1)	-
	*Rattus rattus*	Ingenio	6	3	1	0	3	0	-	3
Total			93	36	3	6	27	6	9	16

^1^ A sample was amplified by a nested PCR using generic primers, but the obtained nucleotide sequence corresponded to organisms other than microsporidia. ^2^ A sample from *M. musculus* showed spore-compatible structures with *Ent. bieneusi* and *Encephalitozoon* spp. under IFAT.

## Data Availability

All the sequences obtained in the present study are publicly available at GenBank.

## Supplementary Materials

The following supporting information can be downloaded at: https://www.mdpi.com/article/10.3390/ani15121695/s1. Table S1: Evaluation of the differences in the occurrence of *Enterocytozoon bieneusi* and microsporidia in wild rodents according to the genus and host species, sex, and island of origin variables. A *p*-value < 0.05 was considered statistically significant. Table S2: Undetermined microsporidia species detected in wild rodents from Canary Islands, Spain.
