# Supplementary material for: Microsporidia in Rodents—Mus musculus, Rattus norvegicus, and Rattus rattus—A Public Health Concern in the Canary Islands, Spain
Source: Animals (Basel). 2025 Jun 8;15(12):1695. doi: 10.3390/ani15121695 (PMC12189156; doi:10.3390/ani15121695)
Supplement: Supplementary file 1 [file animals-15-01695-s001.zip › Table S1 - Statistical results.pdf]

Supplementary Material

Table S1a – Evaluation of the differences in the occurrence of *Enterocytozoon bieneusi* in wild rodents according to the genus and host species, sex and island of origin variables. A *p*-value < 0.05 was considered statistically significant.

Variable		*Enterocytozoon bieneusi*			
**Sex (*n*)**		**Positive (*n*)**	**Negative (*n*)**	**Occurrence (%)**	***p*-value**
Mus musculus	Female (28)	3	25	10.7%	0.324
	Male (22)	1	21	4.5%	
	ND (4)	1	3	25.0%	
Rattus rattus	Female (16)	1	15	6.25%	0.999
	Male (14)	1	13	7.1%	
	ND (5)	0	5	0.0%	
Rattus norvegicus	Female (3)	2	1	66.6%	1
	Male (1)	0	1	0.0%	
**Location (*n*)**		**Positive (*n*)**	**Negative (*n*)**	**Occurrence (%)**	***p*-value**
La Gomera (66)		7	59	10.6%	1
Gran Canaria (27)		2	25	7.4%	
**Host species (*n*)**		**Positive (*n*)**	**Negative (*n*)**	**Occurrence (%)**	***p*-value**
Mus musculus (54)		5	49	9.2%	0.065
Rattus rattus (35)		2	33	5.7%	
Rattus norvegicus (4)		2	2	50.0%	
**Host genus (*n*)**		**Positive (*n*)**	**Negative (*n*)**	**Occurrence (%)**	***p*-value**
Mus (54)		5	49	9.3%	1
Rattus (39)		4	35	10.3%	

Table S1b – Evaluation of the differences in the occurrence of microsporidia in wild rodents according to the genus and host species, sex and island of origin variables. A *p*-value < 0.05 was considered statistically significant.

**Variable**		**Microsporidia**			
**Sex (*n*)**		**Positive (*n*)**	**Negative (*n*)**	**Occurrence (%)**	***p*-value**
Mus musculus	Female (28)	4	24	14.3%	0.198
	Male (22)	5	17	22.7%	
	ND (4)	2	2	50.0%	
Rattus rattus	Female (16)	6	10	37.5%	0.318
	Male (14)	2	12	14.3%	
	ND (5)	2	3	40.0%	
Rattus norvegicus	Female (3)	3	0	100%	1
	Male (1)	1	0	100%	
**Location (*n*)**		**Positive (*n*)**	**Negative (*n*)**	**Occurrence (%)**	***p*-value**
La Gomera (66)		20	46	30.3%	0.3083
Gran Canaria (27)		5	22	18.5%	
**Host species (*n*)**		**Positive (*n*)**	**Negative (*n*)**	**Occurrence (%)**	***p*-value**
Mus musculus (54)		11	43	20.4%	0.00477
Rattus rattus (35)		10	25	28.6%	
Rattus norvegicus (4)		4	0	100%	
**Host genus (*n*)**		**Positive (*n*)**	**Negative (*n*)**	**Occurrence (%)**	***p*-value**
Mus (54)		11	43	20.4%	0.1047
Rattus (39)		14	25	36.0%	

ND = not determined.
