# Supplementary material for: Microsporidia in Rodents—Mus musculus, Rattus norvegicus, and Rattus rattus—A Public Health Concern in the Canary Islands, Spain
Source: Animals (Basel). 2025 Jun 8;15(12):1695. doi: 10.3390/ani15121695 (PMC12189156; doi:10.3390/ani15121695)
Supplement: Supplementary file 1 [file animals-15-01695-s001.zip › Table S2 - Undetermined microsporidia species.pdf]

Supplementary Material

Table S2 – Undetermined microsporidia species detected in wild rodents from the Canary Islands, Spain.

Host (ID)	Island, Municipality	Sequence length (bp)	Query cover (%)	Identity (%)	GenBank reference sequence (accession nº)	Sequence obtained in this study (accession nº)
Rattus rattus (407)	Gran Canaria, Ingenio	312	33%	90.29%	*V. ceranae* (DQ486027)	-
Rattus norvegicus (605)	La Gomera, Hermigua	323	19%	98.33%	*S. perivermis* (KC172651)	-
Rattus rattus (RGB2)	La Gomera, Hermigua	353	17%	91.80%	*E. cuniculi* (L07255)	-
Rattus rattus (410)	La Gomera, Hermigua	311	19%	98.33%	*S. perivermis* (FJ026013)	-
Rattus norvegicus (502P)	La Gomera, Vallehermoso	240	100%	99.58%	*Anncaliia meligethi* (AY894423)	PP873358
Rattus rattus (312)	La Gomera, Hermigua	314	80%	80.08%	*P. cristatellae* (AF484691)	PP883776
		414	31%	89.06%	Microsporidia sp. (OP555065)	PP883777
Mus musculus (507P)	La Gomera, Alajeró	367	22%	93.90%	*E. intestinalis* (OM738339)	PP883778
Rattus rattus (RGB8d)	La Gomera, Vallehermoso	320	77%	79.38%	*P. cristatellae* (AF484694)	PP883779
Mus musculus (309d)	La Gomera, Agulo	207	99%	91.43%	*V. necatrix* (JX213791)	PV061650
Rattus rattus (409)	Gran Canaria, Ingenio	333	71%	100%	Microsporidia sp. (OP555066)	PV061651
Mus musculus (RGB9)	La Gomera, Hermigua	349	99%	92.35%	*T. pampeana* (KM883008)	PV061652
Rattus rattus (RGB12)	La Gomera, Hermigua	330	85%	97.86%	*Microsporidia* sp. (OP555066)	PV061653
Mus musculus (02)	La Gomera, Vallehermoso	364	74%	98.52%	Microsporidia sp. (OP555066)	PV061654

Rattus rattus (08)	Gran Canaria, Ingenio	357	74%	98.49%	Microsporidia sp. (OP555066)	PV061655
Mus musculus (314d)	La Gomera, Hermigua	272	98%	87.64%	*V. lymantriae* (AF141129)	PV061656
Mus musculus (310)	La Gomera, Agulo	235	85%	79.51%	*E. cuniculi* (KX189630)	PV061657
